# Does multidimensional daily information predict the onset of myopia? A 1-year prospective cohort study

**DOI:** 10.1186/s12938-023-01109-8

**Published:** 2023-05-13

**Authors:** Wei Peng, Fei Wang, Shaoming Sun, Yining Sun, Jingcheng Chen, Mu Wang

**Affiliations:** 1 Hefei Institutes of Physical Science, Chinese Academy of Sciences, 350 Shushan Lake Road, Hefei, 230031 Anhui China; 2 University of Science and Technology of China, Hefei, 230026 China; 3 The Second Hospital of Anhui Medical University, Hefei, 230601 China; 4 CAS Hefei Institute of Technology Innovation, Hefei, 230088 China

**Keywords:** Myopia, Prediction, Daily information, Machine learning algorithm, Interpretability

## Abstract

**Purpose:**

This study aimed to develop an interpretable machine learning model to predict the onset of myopia based on individual daily information.

**Method:**

This study was a prospective cohort study. At baseline, non-myopia children aged 6–13 years old were recruited, and individual data were collected through interviewing students and parents. One year after baseline, the incidence of myopia was evaluated based on visual acuity test and cycloplegic refraction measurement. Five algorithms, Random Forest, Support Vector Machines, Gradient Boosting Decision Tree, CatBoost and Logistic Regression were utilized to develop different models and their performance was validated by area under curve (AUC). Shapley Additive exPlanations was applied to interpret the model output on the individual and global level.

**Result:**

Of 2221 children, 260 (11.7%) developed myopia in 1 year. In univariable analysis, 26 features were associated with the myopia incidence. Catboost algorithm had the highest AUC of 0.951 in the model validation. The top 3 features for predicting myopia were parental myopia, grade and frequency of eye fatigue. A compact model using only 10 features was validated with an AUC of 0.891.

**Conclusion:**

The daily information contributed reliable predictors for childhood’s myopia onset. The interpretable Catboost model presented the best prediction performance. Oversampling technology greatly improved model performance. This model could be a tool in myopia preventing and intervention that can help identify children who are at risk of myopia, and provide personalized prevention strategies based on contributions of risk factors to the individual prediction result.

**Supplementary Information:**

The online version contains supplementary material available at 10.1186/s12938-023-01109-8.

## Introduction

Myopia is considered to be a primary public health problem worldwide. According to the WHO, 2.6 billion myopia cases were reported worldwide in 2019 [1]. Myopia is predicted to affect nearly half of the world population by 2050 [2]. In China, the latest government statistics showed that the overall myopia rate of children and adolescents was 52.7% in 2020 [3]. The rate of myopia in primary school students increased rapidly, with an increase of 9.3 percentage points each grade [3].

The importance of myopia prevention is greater than that of treatment. Previous researches have found that earlier onset of myopia increases the risk of high myopia that may cause a series of comorbidities, such as cataract, glaucoma, retinal complications and severe vision loss [4–6]. Moreover, myopia can lead to irreversible visual impairment. Myopia is due to a complex interplay between genetic and environmental factors associated with exposure to the life of a school child [7]. Parental myopia, genetic information, and ocular biometry, such as corneal biomechanical properties, axial length, retinal features and the spherical equivalent refractive error, have been found to be associated with myopia [8–10], and frequently regarded as predictors in prediction models [9, 11–13]. However, the rapid changes in the prevalence of myopia cannot be explained only by genetic reasons, and as a result, scholars indicated the importance of environmental factors [14, 15]. Previous studies have consistently reported that living environment, near work, outdoor time and education were associated factors with myopia [8, 16, 17]. The prediction model incorporating behaviors and environmental factors also had a better performance [18, 19]. However, myopia prediction models only based on easy-collected daily information were few.

Machine learning-based techniques have received increasing attention in a variety of diagnosis and prediction of diseases, such as mental health problems [20], cancer [21] and COVID-19 [22]. Compared with conventional statistical methods, machine learning has shown greater accuracy because of its abilities of fitting high-order and nonlinear relationships between covariates and outcomes [23, 24]. With regard to clinical ophthalmology, various machine learning algorithms have been adopted in the diagnosis of myopia, glaucoma and maculopathy, and the prognosis of intraocular lens implantation [6, 25, 26].

This study aimed to (1) apply machine learning algorithms to establish a model only with easy-collected daily data for the prediction of myopia onset in Chinese school-age children and (2) identify the risk features by interpreting the final model, thereby helping children adjust lifestyles and behaviors to prevent myopia.

## Results

At baseline, 2538 children aged 6–13 years participated in the study and registered their information. After a 1-year follow-up, 174 individuals with incomplete baseline questionnaire data, 28 individuals whose school or residence changed, 92 individuals who lost effective contact, and 23 individuals with eye disease or other health problems were excluded from analyses. Thus, 2221 valid samples were included in the final cohort, 260 (11.7%) of which developed myopia. Comparison of the demographic information difference between non-myopia group versus myopia group in the whole valid dataset is shown in Table 1. Of the 1156 male cases, 119 (10.3%) developed myopia in the following year, and myopia occurred in 141 (13.2%) of the 1065 female participants (*p* < 0.05). Moreover, age and grade were associated with the occurrence of myopia. The mean age of myopia samples was 9.68 ± 1.55, whereas it was 8.98 ± 1.67 for non-myopia samples (*p* < 0.001). With increasing grade level, the rate of myopia onset also increased significantly, and that in grades 1–6 were 2.2%, 6.7%, 13.2%, 15.0%, 17.2%, and 20.1%, respectively.

**Table 1:** Subject demographic information in the final cohort

Variables	Total (mean or n)	Myopia, mean or *n* (%)	Non-myopia, mean or *n* (%)	*P*
All subjects	2221	260 (11.7%)	1961 (88.3%)	
Gender				0.03
Boy	1156	119 (10.3%)	1037 (89.7%)	
Girl	1065	141 (13.2%)	924 (86.8%)	
Region				0.05
Center city	1224	145 (11.8%)	1079 (88.2%)	
Non-center city	997	115 (11.5%)	882 (88.5%)	
Age at baseline	9.06 ± 1.67	9.68 ± 1.55	8.98 ± 1.67	< 0.01
Grade at baseline				< 0.01
1	317	7 (2.2%)	310 (97.8%)	
2	511	34 (6.7%)	477 (93.3%)	
3	469	62 (13.2%),	407 (86.8%)	
4	380	57 (15.0%),	323 (85.0%)	
5	320	55 (17.2%)	265 (82.8%)	
6	224	45 (20.1%)	179 (79.9%)	

The differences in risk factors of myopia between the non-myopia group and myopia group were compared. Variables with statistically significant differences are listed in Table 2, including height, weight, parental myopia, education level of the father, education level of the mother, academic level, hours of homework per day on school days, hours of homework per day on weekends, number of after-school tutoring per week, frequency of extracurricular reading, frequency of visual health education from parents, sitting posture during learning, frequency of lying down reading, frequency of feeling eye fatigue, time of going to sleep at night, frequency of fish intake in the diet, performing Chinese eye exercises regularly, frequency of using electronic devices after turning off the lights at night, the most frequent place to go on weekends, joining sports training teams, main exercise content, hours of outdoor activities per day on school days, and hours of outdoor activities per day on weekends. These 23 factors were associated with the occurrence of myopia in our study. The distribution of the other 15 variables showed no significant differences between the two groups in univariate analysis, namely, the intensity of parents’ requirements for their children’s studies, frequency of class seat exchange, knowledge about eyesight protection, lighting during learning, taking afternoon nap, sleep duration, being choosy in food, frequency of vegetables intake, frequency of bean products intake, hours of using electronic devices per day on school days, hours of using electronic devices per day on weekends, taking breaks during near work, taking a programming class (or courses using computers), number of physical education classes at school per week, and number of physical activities per week. Considering the potential interaction between 23 statistically significant factors, we further calculated the variance expansion factor (VIF) to diagnose the collinearity. The result showed that the VIF for height was the highest, but only 2.58. Thus, all 23 factors did not exist the serious multiple collinearity problems, although partial variables were not mutually independent.

**Table 2:** Univariate analysis of associated factors with the myopia onset

Variable	Total, mean or *n*	Myopia, mean or *n* (%)	Non-myopia, mean or *n* (%)	*P*
Height (cm)	138.76 ± 11.80	143.43 ± 10.56	138.14 ± 11.82	< 0.01
Weight (kg)	34.24 ± 10.29	36.92 ± 9.64	33.88 ± 10.32	< 0.01
Parental myopia				< 0.01
None	1235	98 (7.9%)	1137 (92.1%)	
Father	347	56 (16.1%)	291 (83.9%)	
Mother	421	62 (14.7%)	359 (85.3%)	
Both parents	218	44 (20.2%)	174 (79.8%)	
Education level of the father				0.04
Doctor or master	37	9 (24.3%)	28 (75.7%)	
Bachelor	448	57 (12.7%)	391 (87.3%)	
Below bachelor	1736	194 (11.2%)	1542 (88.8%)	
Education level of the mother				0.01
Doctor or master	26	6 (23.1%)	20 (76.9%)	
Bachelor	330	51 (15.5%)	279 (84.5%)	
Below bachelor	1865	203 (10.9%)	1662 (89.1%)	
Academic level				0.02
Unqualified (grade D)	313	25 (8.0%)	288 (92.0%)	
Qualified (grade C)	798	88 (11.0%)	710 (89.0%)	
Good (grade B)	844	105 (12.4%)	739 (87.6%)	
Excellent (grade A)	266	42 (15.8%)	224 (84.2%)	
Hours of homework per day on school days				0.03
< 1 h	264	20 (7.6%)	244 (92.4%)	
1–2 h	972	105 (10.8%)	867 (89.2%)	
2–3 h	664	89 (13.4%)	575 (86.6%)	
> 3 h	321	46 (14.3%)	275 (85.7%)	
Hours of homework per day on weekends				< 0.01
< 1 h	215	19 (8.8%)	196 (91.2%)	
1–2 h	729	63 (8.6%)	666 (91.4%)	
2–3 h	723	91 (12.6%)	632 (87.4%)	
> 3 h	554	87 (15.7%)	467 (84.3%)	
Number of after-school tutoring per week				< 0.01
0	851	63 (7.4%)	788 (92.6%)	
1–2 times	1034	135 (13.1%)	899 (86.9%)	
3–4 times	262	49 (18.7%)	213 (81.3%)	
> 4 times	74	13 (17.6%)	61 (82.4%)	
Frequency of extracurricular reading				< 0.01
Never	34	4 (11.8%)	30 (88.2%)	
Sometimes	963	89 (9.2%)	874 (90.8%)	
Often	950	123 (12.9%)	827 (87.1%)	
Always	274	44 (16.1%)	230 (83.9%)	
Frequency of visual health education from parents				< 0.01
Always	296	48 (16.2%)	248 (83.8%)	
Often	796	112 (14.1%)	684 (85.9%)	
Sometimes	860	76 (8.8%)	784 (91.2%)	
Never	269	24 (8.9%)	245 (91.1%)	
Sitting posture during learning				
Correct	1581	149 (9.4%)	1432 (90.6%)	
Incorrect	640	111 (17.3%)	529 (82.7%)	
Frequency of lying down reading				< 0.01
Always	48	7 (14.6%)	41 (85.4%)	
Often	340	63 (18.5%)	277 (81.5%)	
Sometimes	1317	147 (11.2%)	1170 (88.8%)	
Never	516	43 (8.3%)	473 (91.7%)	
Frequency of feeling eye fatigue				< 0.01
Never	692	32 (4.6%)	660 (95.4%)	
Sometimes	1313	152 (11.6%)	1161 (88.4%)	
Often	187	65 (34.8%)	122 (65.2%)	
Always	29	11 (37.9%)	18 (62.1%)	
Time of going to sleep at night				0.02
Before 9 o'clock	433	33 (7.6%)	400 (92.4%)	
9–10 o'clock	1437	175 (12.2%)	1262 (87.8%)	
10–11 o'clock	330	50 (15.2%)	280 (84.8%)	
11–12 o'clock	15	1 (6.7%)	14 (93.3%)	
After 12 o'clock	6	1 (16.7%)	5 (83.3%)	
Frequency of fish intake in the diet				0.03
Never	294	43 (14.6%)	251 (85.4%)	
Sometimes	964	108 (11.2%)	856 (88.8%)	
Often	805	82 (10.2%)	723 (89.8%)	
Always	158	27 (17.1%)	131 (82.9%)	
Performing the Chinese eye exercises regularly				< 0.01
Yes	1719	180 (10.5%)	1539 (89.5%)	
No	502	80 (15.9%)	422 (84.1%)	
Frequency of using electronic devices after turning off the lights at night				< 0.01
Always	11	0 (0.0%)	11 (100.0%)	
Often	23	8 (34.8%)	15 (65.2%)	
Sometimes	410	59 (14.4%)	351 (85.6%)	
Never	1777	193 (10.9%)	1584 (89.1%)	
The most frequent place to go on weekends				< 0.01
Sports venues	407	40 (9.8%)	367 (90.2%)	
Leisure or entertainment places	333	23 (6.9%)	310 (93.1%)	
Learning places	643	105 (16.3%)	538 (83.7%)	
Staying at home	838	92 (11.0%)	746 (89.0%)	
Joining sports training teams				0.03
Yes	744	72 (9.7%)	672 (90.3%)	
No	1477	188 (12.7%)	1289 (87.3%)	
Main exercise content				0.04
Strength training	6	2 (33.3%)	4 (66.7%)	
Jogging	423	58 (13.7%)	365 (86.3%)	
Ball games	473	42 (8.9%)	431 (91.1%)	
Uncertain	1319	158 (12.0%)	1161 (88.0%)	
Hours of outdoor activities per day on school days				< 0.01
Less than 1 h	1079	155 (14.4%)	924 (85.6%)	
1–2 h	792	81 (10.2%)	711 (89.8%)	
2–3 h	250	18 (7.2%)	232 (92.8%)	
More than 3 h	100	6 (6.0%)	94 (94.0%)	
Hours of outdoor activities per day on weekends				< 0.01
Less than 1 h	599	81 (13.5%)	518 (86.5%)	
1–2 h	892	119 (13.3%)	773 (86.7%)	
2–3 h	490	44 (9.0%)	446 (91.0%)	
3–4 h	119	9 (7.6%)	110 (92.4%)	
More than 4 h	121	7 (5.8%)	114 (94.2%)	

**Fig. 1:**
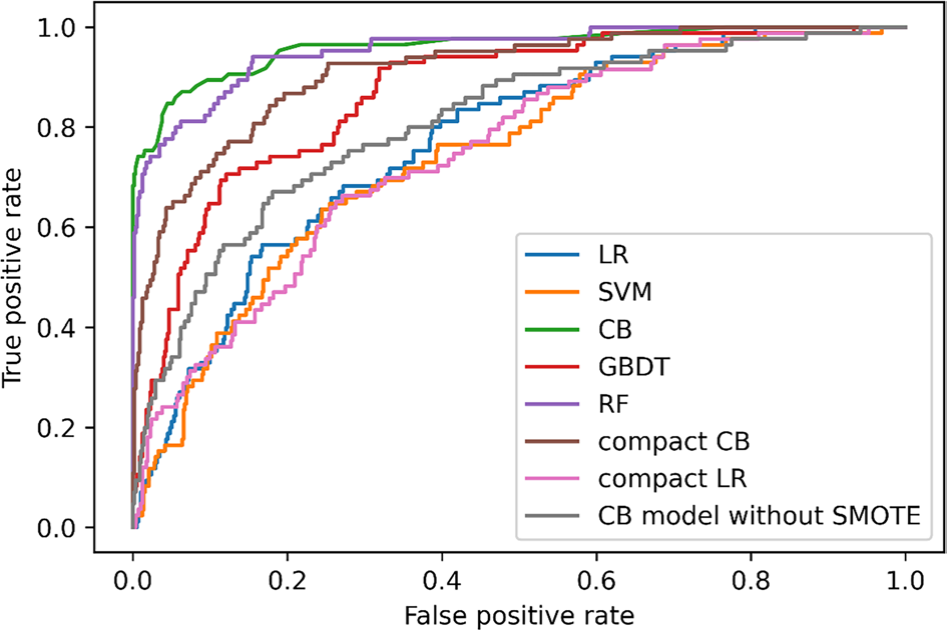
ROC curves for each algorithm in the test set. *LR* Logistic Regression, *SVM* Support Vector Machines, *RF* Random Forest, *GBDT* Gradient Boosting Decision Tree, *CB* CatBoost, *SMOTE* Synthetic Minority Over-sampling Technique

The final 26 statistically significant variables (23 listed in Table 2 and demographic factors, gender, age, grade) were entered into each model as predictors. After using the SMOTE in the training set, the size of the low-portion group (myopic group) was expanded and the ratio of myopic to nonmyopic cases was 1:1. In rows 1–5 of Table 3, the metrics of five algorithms based on the model validation were compared in terms of precision, recall, F1-score and AUC. The precision values of these five models (LR, SVM, GBDT, RF and CB) were 0.892, 0.883, 0.887, 0.934, and 0.953, and the recall values were 0.014, 0.013, 0.221, 0.494, and 0.639, respectively. With regard to F1-score, CB had the highest value (0.774). ROC curves of the five models are displayed in Fig. 1. Among the five models, CB also had the highest AUC value (0.951), whereas that of SVM was lowest (0.647). After overall consideration of the predicting performance, we selected the model using the CB algorithm over the others to perform further analysis in the present study.

**Table 3:** Model performance using five algorithms in test set

Model	Precision	Recall	F1-score	AUC
LR	0.892	0.014	0.027	0.739
SVM	0.883	0.013	0.025	0.647
GBDT	0.887	0.221	0.336	0.865
RF	0.934	0.494	0.651	0.935
CB	0.953	0.639	0.774	0.951
CB (without the SMOTE)	0.889	0.133	0.213	0.763
CB (the compact model)	0.905	0.320	0.432	0.891
LR (the compact model)	0.887	0.133	0.211	0.692

The 10 predictors used on the compact model: parental myopia, grade, frequency of feeling eye fatigue, height, weight, frequency of visual health education from parents, academic level, number of after-school tutoring per week, frequency of fish intake in the diet and hours of outdoor activities per day on school days

*LR* Logistic Regression, *SVM* Support Vector Machines, *RF* Random Forest, *GBDT* Gradient Boosting Decision Tree, *CB* CatBoost

Considering the effect of the SMOTE on modeling, we used the original data without the SMOTE to train a new model and test it in the same test dataset. As shown in Table 3 and Fig. 1, the precision, recall, F1-score, and AUC value of the CB model without the SMOTE in the test set were 0.889, 0.133, 0.213, and 0.763, respectively, which have dropped significantly.

**Fig. 2:**
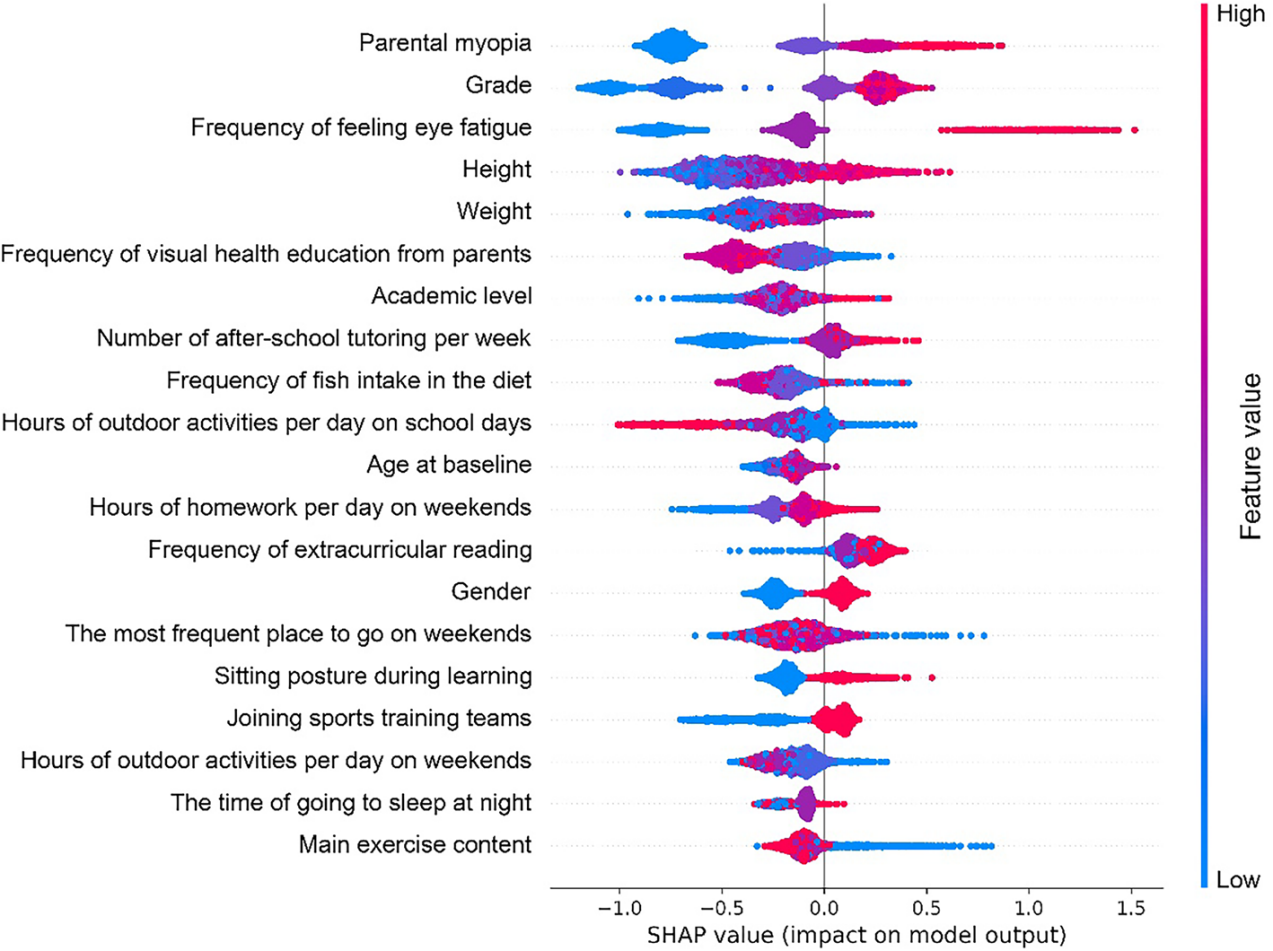
Global view of feature impact of the Catboost model based on the SHapley Additive exPlanations (SHAP) values. The plot sorts features in descending order of their impact on the model output. Each dot in the visualization represents one datapoint of a feature. The color represents the feature value: high value in red and low value in blue

As shown in Fig. 2, SHAP values of the CB model were calculated and plotted to show the distribution of the effects of each feature on the model output. The features were ranked in descending order of their effects. The top 10 features were as follows: parental myopia, grade, frequency of feeling eye fatigue, height, weight, frequency of visual health education from parents, academic level, number of after-school tutoring per week, frequency of fish intake in the diet and hours of outdoor activities per day on school days, demonstrating their importance in predicting myopia. Thus, a compact CB model was built and tested on the basis of these top 10 features in SHAP values. As shown in the last two rows of Table 3 and Fig. 1, this compact model had a slightly decreased F1-score (0.432 vs. 0.774) and AUC value (0.891 vs. 0.951) compared with the full model, but it still outperformed traditional logistic regression model based on 10 predictors (AUC, 0.891 vs. 0.692), and even the logistic regression model with 26 predictors (AUC, 0.877 vs. 0.739).

**Fig. 3:**
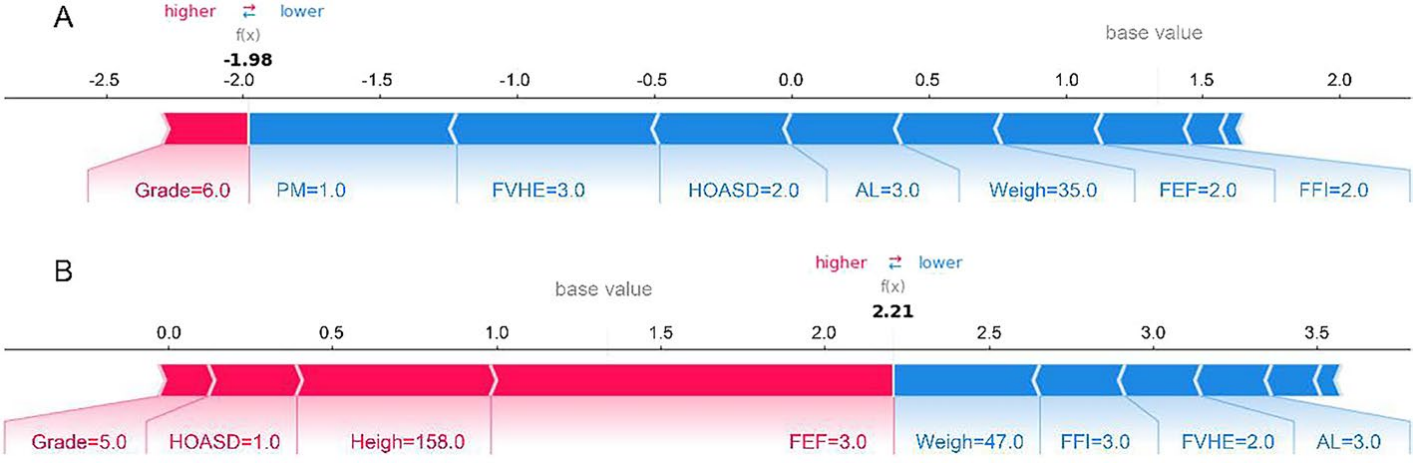
The prediction results and individual force views of feature impacts for two specific instances. The base value is the average value of the prediction model of myopia onset. The *f*(*x*) is the output value of the model. The bars in red and blue represent risk and protective effects, respectively. Longer bars indicate greater feature importance. Figures **A** and **B** show the cause of low-risk and high-risk instance, respectively. *PM* parental myopia, *FVHE* frequency of visual health education from parents, *HOASD* hours of outdoor activities per day on school days, *AL* academic level, *FEF* frequency of eye fatigue, *FFI* frequency of fish intake in the diet

Figure 2 shows the global view of features ranking. In addition, individual force views of the successful prediction results for two specific instances are shown in Fig. 3. For the case shown in Fig. 3A, the output value of the model was a negative number that indicated that this case would have a low risk of myopia onset in the following year. Parents without myopia (PM = 1.0), visual health education from parents (FVHE = 3.0), and 1–2 h of outdoor activities per day on school days (HOASD = 2.0) would play major protective roles. As shown in Fig. 3B, our model predicted that this case would be myopia if he/she kept the current state of lifestyles and behaviors in the following year. Frequently feeling eye fatigue (FEF = 3.0) was the most important risk signal, and the contribution of other protective factors was weak.

## Discussion

In the present study, we investigated factors associated with myopia and observed the incidence of myopia 1 year later in the sample of primary school students. By using machine learning algorithms, the predictive models for the incidence of myopia were developed and validated. As previous studies presented, the age of onset of myopia was associated with the likelihood that a child will experience progression to vision-threatening levels of myopia [4, 5, 35]. The recent International Myopia Institute Report has pointed out that practitioners and parents should be active in addressing myopia onset and progression at as young an age as possible [8, 36]. As a guidance tool, this model can effectively identify children who are at risk of myopia by investigating easy-collected daily information, and interpret the impact of risk factors on the prediction result at the individual level, which would help provide the accurate suggestions of myopia prevention.

Individual daily information covers a large number of factors related to the occurrence of myopia. Gender, grade, parental myopia, education level of parents, time outdoors, etc., were associated with the myopia occurrence and these factors have been consistently reported by previous studies [8, 37, 38]. Moreover, we observed some risk factors with varying strengths of the association with myopia. Our results showed that height and weight were associated with myopia, but the association between height and myopia was not found by Terasaki et al. [39]. The results showed that children who regularly performed Chinese eye exercises were less likely to be myopic than those who did not. However, whether these exercises could make a difference remains unclear. A cross-sectional survey by Huang et al. did not indicate the link between Chinese eye exercises and myopia [40]. Furthermore, the relationship between education and myopia has always been a hot topic. In this study, we indicated the effect of educational pressure with regard to academic level, the amount of homework per day, and the number of after-school tutoring per week on myopia, which were consistent with the hypothesis [8, 17]. In addition, some factors, such as the subjective feeling of eye fatigue, dietary habits and the preference for different types of sports were associated with the myopia occurrence and these factors were rarely discussed in previous studies. The pathogenesis of eye fatigue is understood to result from a complex interplay of visual/environmental conditions and physiological factors that modulate the visually guided refraction. Visual display terminal work, long-term near work, poor indoor lighting environment may cause the eye fatigue [41, 42]. We also found that playing ball games can reduce the incidence of myopia compared with strength training and jogging.

With the clarity of risk factors, the prediction model of myopia has also been widely concerned. Our compact model including only 10 non-ocular features had a high prediction performance even better than some models including ocular biometry and genetic information [19, 43, 44]. Wong et al. developed models based non-cycloplegic SE, axial length and positive relative accommodation, with AUC values of 0.64, 0.62, and 0.66, respectively, and their combination with age, gender and parental myopia only achieved an AUC of 0.74 [43]. A model only with non-ocular features, including parental myopia, number of books read per week, time spent reading, participation in sports, time spent outdoors, and ethnicity, was built to predict myopia incidence in 6- to 9-year-old children with an AUC of 0.63 [18]. In addition, the Collaborative Longitudinal Evaluation of Ethnicity and Refractive Error Study showed that the AUC value of the model using only the demographic data ranged from 0.58 to 0.68 [9].

Notably, the number of environmental risk factors studied in previous models was limited, which may affect the performance of the models. Furthermore, we found that resolving the class imbalance by using the SMOTE in the model development greatly improve the model performance. The results showed that the CB model that did not use the SMOTE only achieved an AUC of 0.763, which has no clear advantage over other models using only non-ocular features [9, 18], and was significantly lower than that of models that included ocular biometry [44]. As shown in Table 3, although an AUC of 0.763 and a precision value of 0.889 may be acceptable, the recall value and F1-score almost failed. The recall value of 0.133 from the model validation indicated that the model development overfitted the data in the nonmyopic student group and resulted in prediction biased towards the nonmyopic students. With the help of the SMOTE, the recall value increased from 0.133 to 0.639. Thus, the oversampling technique avoided the biased results. Additionally, the F1-score calculated by the precision and the recall values should be considered as an important indicator of the model performance and not be ignored.

In this study, results have demonstrated that machine learning models (GBDT, RF and CB) were better than the conventional logistic regression method in the myopia prediction. Machine learning models excel in the analysis of complex signals in data-rich environments [24]. In terms of the current dataset, the main reasons may be as follows: (1) we hypothesize that the dataset with imbalanced classes is a key factor. Although we have used the oversampling technology to address this issue, the logical regression model was still very sensitive and showed a very low F1-score on the test dataset. This indicates that the accuracy and generalization ability of the logistic regression model were weak for the original dataset with imbalanced classes. (2) Our model was a high-dimensional space with 26 variables. The machine learning model has a stronger power to process large training data with high dimensionality better than the logistic regression model. (3) The machine learning model can automatically capture the complex relationship between covariates and outcomes, such as high-order and nonlinear relationships. (4) Compared with regression-based method, the machine learning model can also improve predictive accuracy by exploiting complex interactions between predictors [6].

Furthermore, the CatBoost model presented the advantages, with the highest F1-score and AUC value. Considering the better usability and lower socioeconomic burden, we developed a compact model with 10 features, whose performance was slightly reduced compared with the full Catboost model, but still better than the compact logical regression model, and even the full logical regression model. As a new member of the family of machine learning techniques, the Catboost has shown important value and potential in the wide variety of fields since its debut in December 2018 [33, 45]. In machine learning modeling, categorical features are usually preprocessed to convert categories to their target statistics which may cause target leakage and prediction shift [46]. The Catboost algorithm uses the ordered target statistics encoding to explicitly operate with categorical features, and avoid prediction shift through an ordered boosting technique in training [33, 46]. Thus, Catboost performs well for categorical variables in the data. Since there were a large number of categorical variables in our dataset, the Catboost model achieved better performance than other machine learning models. Moreover, the SHAP technique has been successfully applied in our final compact model to explain the outcome of the prediction. At the instance level, as shown in Fig. 3, the individual force view can explicitly illustrate the combined effect of risk factors and protective factors on myopia onset, which provides clear prevention strategies and makes our model clinically interpretable.

Our study has several limitations. First, behaviors related to risk factors of myopia, such as extracurricular reading, the time of going to sleep and time outdoors, may have changed during the course of the study. Second, the screening procedure at baseline did not include measurement of cycloplegic refraction. At 1-year visit, only children with low visual acuity were asked for further examination by using cycloplegic refraction. Those who achieved normal VA could still be myopes by cycloplegic refraction.

## Conclusions

Based on easy-collected daily information, a prediction model of myopia onset was presented, with the satisfied performance. The outcome of the model and visual interpretability of feature impacts could be used to identify those at risk of myopia onset and provide corresponding preventive advice, which may help children timely make valid adjustments to prevent or slow the early onset of myopia.

## Methods

### Study population

This school-based prospective cohort study was conducted in Anhui, China, in February, 2021. Five primary schools were selected as pilot schools, three of which were from center cities, and two schools were from non-center cities. The inclusion criteria of students were as follows: (1) children did not use any myopia control treatment; (2) participants had no other oculopathies or refractive errors, such as hyperopia, astigmatism, strabismus and glaucoma; (3) individuals could be visited in the next year (from February 2021 to February 2022); (4) participating students had no plans to transfer, and participating families had no plans to move.

### Data collection and definition of variables

At baseline, all children underwent visual acuity tests using the standard five-point logarithmic visual acuity E chart [27]. Poor vision was defined as uncorrected visual acuity (VA) < 5.0 (Snellen equivalent 20/20) in either eye. Similar to the previous study [9], we classified those children who had normal visual acuity, not used any myopia control treatment, and had no ophthalmic history, as screening nonmyopic. Then, all children who were nonmyopic at baseline and their parents completed a structured questionnaire, which was developed from different literature associated with risk factors of myopia [8, 9, 15, 17, 28, 29]. A total of 42 independent variables were collected on the basis of five aspects, including demographic information, parental education and their myopia, daily lifestyles and behaviors, educational burden, and outdoor activities (Additional file 1).

One year later (February 2022), we evaluated the incidence of myopia in children of the initial cohort. Visual acuity tests were performed again by using the standard logarithmic visual acuity chart. Individuals with VA worse than 5.0 were refracted with cycloplegic refraction by ophthalmologists using 1% cyclopentolate eye drop. Children who had already been diagnosed with myopia (cycloplegic refractive state) during this follow-up year would be considered to be myopic and not attend the myopia assessment in the follow-up test. Myopia at person level was defined as spherical equivalent < −0.5 diopter (D) in either eye.

### Statistical analysis

All values were expressed as means ± standard deviation for continuous variables or as counts and percentages for categorical variables. Differences in the distribution of variables between the non-myopia group and myopia group were assessed using the Chi-square test for categorical variables, Student’s *t*-test for normally distributed continuous variables, and nonparametric test for non-normally distributed continuous variables. Statistically significant features with a *p*-value less than 0.05 in univariate analysis were set as initial predictors. These analyses were performed by using the Statistical Package for Social Sciences (SPSS v22.0).

### Machine learning algorithms and modeling

First, we randomly split our data into a training set (70% of the sample) and a test set (30%). Then, the Synthetic Minority Over-sampling Technique (SMOTE) was used to resolve the class imbalance in the training set. The SMOTE expanded sample size of the low-portion group (myopic group) by identifying an individual in the low-portion group and then finding its k-nearest neighbors. A data set with the class balance can avoid overfitting the data to the high-portion group and improve the classification performance. In addition, fivefold cross-validation was applied in the training set to avoid overfitting. Based on this method, the entire training set was further divided into five subsets, and training was repeated for five rounds. Of the five subsets, a single subset was used for validating, and the remaining four subsets were used for training each round.

With regard to learning algorithms, we selected four classical machine learning algorithms, namely Random Forest (RF), Support Vector Machines (SVM), Gradient Boosting Decision Tree (GBDT) and CatBoost (CB), as well as the standard statistical method, Logistic Regression (LR). The SVM algorithm classifies the samples by transforming training data into a high-dimensional feature space, and then solving the maximum margin hyperplane in this multidimensional space [30]. The RF, GBDT and CB are all ensemble supervised learning method and use decision trees as the base weak learner. The RF comprises multiple decision trees which are trained on the data subsets or with the feature subspace. Each tree calculates the results and is combined together in parallel to generate a strong learner [31, 32]. The GBDT fits a sequence of such decision trees in series. It minimizes the residual using gradient descents and uses residual as the target for the iteration training [31, 33]. The CB is a modification of GBDT, and brings two innovations: ordered Target Statistics and Ordered Boosting [33].

Based on the test dataset, we used the Precision, Recall, F1-score values and area under the receiver operating characteristic (ROC) curve (AUC) to evaluate the predictive performance of each model. The model with the best prediction outcomes in the validation was adopted for further analysis:

$$\mathrm{Precision}=\frac{\mathrm{TP}}{\mathrm{TP}+\mathrm{FP}},$$

$$\mathrm{Recall}=\frac{\mathrm{TP}}{\mathrm{TP}+\mathrm{FN}},$$

$$F1=2\times \frac{\mathrm{Precision}\times \mathrm{Recall}}{\mathrm{Precision}+\mathrm{Recall}}.$$

TP, true positive, indicating the positive class is predicted as the number of positive classes; FP, false positive, indicating the negative class is predicted as the number of positive classes; FN, false negative, indicating the positive class is predicted as the number of negative classes.

In order to evaluate the importance of features and obtain interpretations of the features from the prediction model results, we applied the Shapley Additive exPlanations (SHAP) technique. SHAP connects the game theoretic approach with local explanations by using classic Shapley values from the game theory and their related extensions [34]. It assigns each feature an importance value for a particular prediction and provides a global view of feature ranking and individual force views [22]. Finally, a feature subset was selected on the basis of the feature importance to construct a compact model.

To develop modeling algorithms, we used the scikit-learn library, a machine learning toolkit based on Python language. Python 3.8.10 and Jupyter Notebook were used as development environments.

## Supplementary Information

**Additional file 1: **Registration of Baseline Information and Questionnaire for Risk Factors of Myopia.

## Data Availability

The datasets analyzed during the current study are available from the corresponding author on reasonable request.
