# Supplementary material for: Does multidimensional daily information predict the onset of myopia? A 1-year prospective cohort study
Source: Biomed Eng Online. 2023 May 13;22:45. doi: 10.1186/s12938-023-01109-8 (PMC10182351; doi:10.1186/s12938-023-01109-8)
Supplement: Supplementary file 1 — Additional file 1: Registration of Baseline Information and Questionnaire for Risk Factors of Myopia. [file 12938_2023_1109_MOESM1_ESM.docx]

**Registration of Baseline Information and Questionnaire for Risk Factors of Myopia**

This questionnaire is for investigating factors associated with myopia among primary school students in Anhui. This work will aid in studying on the prediction of myopia in children. Thanks for your participation, thank you!

**Demographic Information**

1. Name (ID):

2. Age:

3. Gender:

A. male B. female

4. Grade:

A. grade 1 B. grade 2 C. grade 3

D. grade 4 E. grade 5 F. grade 6

5. City: A. center city (Hefei) B. non-center city

6. School:

7. Height (cm) Weight (kg)

**Parental Education and Their Myopia**

8. Parental myopia or not

A. none B. only father C. only mother D. both

9. Education level of the father

A. doctor or master B. bachelor C. below bachelor

10. Education level of the mother

A. doctor or master B. bachelor C. below bachelor

**Educational Burden**

11. Children’s academic level at school (based on the last exam results)

A. excellent (grade A) B. good (grade B)

C. qualified (grade C) D. unqualified (grade D)

12. Parents’ requirements for children’s academic level

A. very high B. high

C. general D. low

13. Hours of children’s homework per day on school days

A. less than 1 hour B. 1–2 hours C. 2–3 hours D. more than 3 hours

14. Hours of children’s homework per day on weekends?

A. less than 1 hour B. 1–2 hours C. 2–3 hours D. more than 3 hours

15. Number of children’s after-school tutoring per week

A. none B. 1–2 times C. 3–4 times D. more than 4 times

16. Whether children take a programming class (or courses using computers)?

A. yes B. no

**Daily Lifestyles and Behaviors**

17. Whether lighting during learning is good?

A. yes B. no

18. Frequency of extracurricular reading

A. never B. sometimes C. often D. always

19. Whether children are knowledgeable about eyesight protection?

A. yes B. no

20. Whether parents often carry out visual health education?

A. never B. sometimes C. often D. always

21. Whether children’s sitting posture is correct during learning?

A. yes B. no

22. Frequency of class seat position exchange at school

A. once a week B. once every two weeks C. once a month

C. once a semester D. none (only individual students)

23. Frequency of lying down reading

A. never B. sometimes C. often D. always

24. How often do children take a break during near work?

A. less than 0.5 hour B. 0.5–1 hour C. 1–2 hours

C. 2–3 hours D. > 3 hours

25. Frequency of feeling eye fatigue

A. never B. sometimes C. often D. always

26. Whether children take afternoon nap?

A. yes B. no

27. Time of going to sleep at night

A. before 9 o'clock B. 9–10 o'clock C. 10–11 o'clock

D. 11–12 o'clock E. after 12 o'clock

28. Children’s sleep duration at night

A. less than 6 hours B. 6–8 hours

C. 8–10 hours D. more than 10 hours

29. Whether children are choosy in food?

A. yes B. no

30. Frequency of vegetables intake in the diet

A. never B. sometimes C. often D. always

31. Frequency of vegetables intake in the diet

A. never B. sometimes C. often D. always

32. Frequency of bean products intake in the diet

A. never B. sometimes C. often D. always

33. Whether children perform the Chinese eye exercises regularly?

A. yes B. no

34. Hours of using electronic devices per day on school days

A. less than 1 hour B. 1–2 hours

C. 2–3 hours D. more than 3 hours

35. Hours of using electronic devices per day on weekends

A. less than 1 hour B. 1–2 hours

C. 2–3 hours D. more than 3 hours

36. Frequency of using electronic devices after turning off the lights at night

A. never B. sometimes C. often D. always

37. The most frequent place to go on weekends

A. sports venues B. leisure or entertainment places

C. learning places D. staying at home

**Outdoor Activities**

38. Number of physical education classes at school per week

A. more than 4 classes B. 3–4 classes C. 1–2 classes

39. Joining sports training teams

A. yes B. no

40. Number of physical activities except physical education classes per week

A. more than 4 times B. 3–4 times

C. 1–2 times D. none

41. Children’s main exercise content

A. strength training B. jogging C. ball games D. uncertain

42. Hours of outdoor activities per day on school days

A. less than 1 hour B. 1–2 hours

C. 2–3 hours D. more than 3 hours

43. Hours of outdoor activities per day on weekends

A. less than 1 hour B. 1–2 hours C. 2–3 hours

D. 3–4 hours E. more than 4 hours
